# Association of the *ABCG2* rs2231142 variant with the Framingham Cardiovascular Disease Risk score in the Taiwanese population

**DOI:** 10.1016/j.heliyon.2024.e37839

**Published:** 2024-09-11

**Authors:** Chun-Kang Lee, I-Chieh Chen, Hsueh-Ju Lin, Ching-Heng Lin, Yi-Ming Chen

**Affiliations:** a Division of Gastroenterology and Hepatology, Taichung Veterans General Hospital, Taichung, Taiwan; b Department of Medical Research, Taichung Veterans General Hospital, Taichung, Taiwan; c Department of Public Health, College of Medicine, Fu Jen Catholic University, New Taipei City, Taiwan; d Division of Allergy, Immunology and Rheumatology, Taichung Veterans General Hospital, Taichung, Taiwan; e Department of Post-Baccalaureate Medicine, College of Medicine, National Chung Hsing University, Taichung, Taiwan; f Graduate Institute of Clinical Medicine, National Chung Hsing University, Taichung, Taiwan; g School of Medicine, College of Medicine, National Yang Ming Chiao Tung University, Taipei, Taiwan

**Keywords:** Coronary heart disease, *ABCG2* rs2231142, Uric acid, Predictors, Single-nucleotide polymorphism, Precision healthcare

## Abstract

**Background:**

Serum uric acid (SUA) is an important predictor of cardiovascular events and mortality. The *ABCG2* rs2231142 variant (TT genotype) is associated with hyperuricemia (HUA), but the relationship between *ABCG2* gene polymorphisms and coronary artery disease (CAD) risk is poorly elucidated. We investigated the association between *ABCG2* rs2231142 genetic variants and the Framingham Risk Score for Cardiovascular Disease (FRS-CVD) in a Taiwanese population.

**Methods:**

This cross-sectional study enrolled 139,508 Taiwanese participants aged 30–70 years based on data from the Taiwan Biobank (TWB) database that was obtained from questionnaires, laboratory investigations, anthropometry, and Affymetrix TWB genome-wide single-nucleotide polymorphism (SNP) chip data analysis. The association between *ABCG2* rs2231142 and FRS-CVD risk was evaluated using logistic regression analysis.

**Results:**

Compared to those with the GG genotype, participants with the *ABCG2* rs2231142 TT genotype had a significantly lower systolic blood pressure, smoking rate, body mass index, triglyceride level, waist circumference, waist–hip ratio, and body fat percentage, but had higher high-density lipoprotein cholesterol level. Despite the same FRS-CVD score, participants with TT genotypes had higher SUA. Even with the same SUA, TT carriers had a lower FRS-CVD than GT and GG carriers. Participants with the TT genotype had significantly lower CVD risk, particularly female participants with HUA and BMI <27 (OR: 0.760, 95 % CI: 0.587–0.985; p = 0.0381) group.

**Conclusion:**

The *ABCG2* rs2231142 TT genotype is associated with a lower FRS-CVD, particularly in non-obese hyperuricemic female individuals. The complicated interplay among genetic variations, metabolic profile, and CVD risk provides insights for precision health.

## Highlights

• *ABCG2* rs2231142 TT genotype is associated with hyperuricemia.
• The TT risk allele is associated with factors within and beyond the Framingham risk score.
• The TT risk allele is independently associated with lower Framingham risk score in non-obese hyperuricemic female population.
• A gender-specific nomogram for predicting Framingham Risk Score by uric acid level and ABCG2 genotype is provided for the Taiwanese population.

## Abbreviations

**APT**: Affymetrix Power Tools
**BMI**: Body mass index
**CAD**: Coronary artery disease
**CI**: Confidence intervals
**CVD**: Cardiovascular disease
**DM**: Diabetes mellitus
**eGFR**: Estimated glomerular filtration rate
**FRS**: Framingham Risk Score
**FRS-CVD**: Framingham Risk Score for Cardiovascular Disease
**GWAS**: Genome-wide association studies
**HUA**: Hyperuricemia
**HWE**: Hardy–Weinberg equilibrium
**HDL-C**: High-density lipoprotein cholesterol
**ICD**: International classification of diseases
**LDL-C**: Low-density lipoprotein cholesterol
**MDRD**: Modification of Diet in Renal Disease
**OR**: Odds ratios
**SBP**: Systolic blood pressure
**SNP**: Single-nucleotide polymorphism
**SUA**: Serum uric acid
**TC**: Total cholesterol
**TG**: Triglyceride
TWB: Taiwan Biobank
**UACR**: Urinary albumin-to-creatinine ratio
**WHR**: Waist–hip ratio

## 1 Introduction

The Framingham Risk Score (FRS) estimates the 10-year risk for coronary artery disease (CAD) using six factors [1]: age, total cholesterol (TC), high-density lipoprotein cholesterol (HDL-C), systolic blood pressure (SBP), diabetes mellitus (DM), and smoking status. In 2008, the FRS was expanded to predict the 10-year risk for various cardiovascular diseases (CVD), including CAD, heart failure, cerebrovascular events, and peripheral artery disease [2]. Despite its widespread use, the global validation of Framingham Risk Score for Cardiovascular Disease (FRS-CVD) risk assessment is limited [3]. Therefore, improving FRS-CVD prediction requires developing accurate risk profiles and incorporating novel genetic factors. Proposed genetic risk scores [4], which include genes like CDKN2B-AS1, PHACTR1, FLT1, VAMP5, IL6R, LPA, LPL, and APOC1 [5], still need verification across different ethnic groups.

A multicenter study showed that, in the Chinese adult population, hyperuricemia (HUA) was associated with multiple major CVD risk factors [6]. HUA contributes to CVD pathogenesis through mechanisms that promote oxidative stress, inflammatory response, insulin resistance, endothelial dysfunction, and endoplasmic reticulum stress [7]. The association of CVD risk and uric acid has been identified not only in the hyperuricemic population but also in the normal serum uric acid (SUA) group [8]. Genome-wide association studies have identified several genes linked to gout and HUA [9]. The most prominent and well-established risk variant is *ABCG2* rs2231142 single nucleotide polymorphism (SNP), a missense SNP that could be causally related to at least 10 % of all gout cases [10].

Recent studies have demonstrated that *ABCG2* rs2231142 variant and the SUA level increased markedly in those with a clustering of CVD risk factors [6,11]. ABCG2, initially recognized as a multidrug resistance protein, is involved in transporting urate, lipids, and other substrates across various tissues [10,12], including the renal tubules, intestines, and liver [13,14]. The ABCG2 rs2231142 variant, located in the nucleotide-binding domain, is crucial for protein stability [15]. The TT genotype specifically reduces ATPase activity and uric acid transport, leading to decreased expression and function of the protein, both in vitro and in vivo [15]. This variant's impaired activity potentially contributes to its impact on cardiovascular disease risk by altering urate metabolism.

To determine whether *ABCG2* rs2231142 variants are associated with the FRS-CVD score, this study investigated the relationship of *ABCG2* genetic variations with the CVD risk scores.

## 2 Materials and methods

### 2.1 Study population

This cross-sectional study was conducted by using data from the Taiwan Biobank (TWB) that collected information and specimens of Taiwanese volunteers from recruitment centers in Taiwan. The community-based database, from Taiwan Academia Sinica, included information collected via a questionnaire, physical examination, blood and urine tests, and experimental data of participants aged 30–70 years without any history of cancer. Detailed information on the program and data access are available from the official website of the TWB (https://taiwanview.twbiobank.org.tw/index) [16]. In total, 139,508 cancer-free participants aged 30–70 years who volunteered to participate in the TWB database were identified. Additionally, the database provided CAD and hyperlipidemia history as well as demographic, biochemical, and Affymetrix TWB 650 K single-nucleotide polymorphism (SNP) chip data. The entire database of TWB was utilized for this study. This study was approved by the ethics committee of Taichung Veterans General Hospital Institutional Review Board (approval no. CE16270B-2). This research was conducted in accordance with the principles of the Declaration of Helsinki and the Good Clinical Practice Guidelines, and the IRB waived the requirement for informed consent because the participants’ data were anonymized prior to analysis.

### 2.2 SNP genotyping

Genotyping using blood DNA samples from TWB participants was conducted by using the genome-wide TWB 2.0 array plate specifically developed for Han Chinese population in Taiwan. TWB 2.0 is a technology platform that has been developed by Affymetrix (Affymetrix, Santa Clara, California, USA) and is applied to the TWB 650 K SNPs array for selected SNPs [17]. The Affymetrix Power Tools (APT; command-line software, Santa Clara, California, USA) was used for genotype calling and to exclude SNPs with low call rates (<99 %) by following a standard quality-control procedure. A *p*-value <1.0 × 10^−5^ for controls and <0.05 for minor allele frequency was used for the Hardy–Weinberg equilibrium (HWE). A total of 653,291 autosomal SNPs passed the quality-control assessment. The data analyzed included information on SNPs of the sex chromosomes and mitochondrial DNA [16].

*ABCG2* rs2231142 was chosen for further analysis because it exhibited the most significant impact on gout and hyperuricemia compared to other SNPs within the *ABCG2* gene [15].

### 2.3 Framingham Risk Score

The primary outcome was the 10-year CVD risk, calculating using the FRS-CVD scores [18], which was based on age, TC, HDL-C, SBP, DM, and smoking status. Patients were stratified into a high-risk group (≥10 % CVD risk, including intermediate and high risk) and a low-risk group for the logistic regression.

### 2.4 Measurement of covariates

The SUA level was measured by the Architect i2000SR Analyzer (Abbott Diagnostics, Abbott Park, Chicago, IL, USA) using the uricase method [19]. Male and female participants with SUA ≥7.0 and ≥ 6.0 mg/dL, respectively, were diagnosed with HUA [20].

Lifestyle and biochemical data were extracted from the TWB dataset. Covariates including in the analysis included: participants' demographic (sex and age), physical examination (body mass index [BMI] and waist circumference), comorbidity (DM), lifestyle (smoking), and blood and urine tests (fasting glucose, creatinine, TC, triglyceride [TG], HDL-C, and low-density lipoprotein cholesterol [LDL-C, all in mg/dL]). Kidney function was assessed by estimated glomerular filtration rate (eGFR, mL/min/1.73 m^2^), calculated by Modification of Diet in Renal Disease (MDRD) formula. Microalbuminuria was determined by using the urinary albumin-to-creatinine ratio (UACR, mg/g). BMI was calculated as the weight (kilograms [kg]) divided by height squared, meters [m^2^]). According to the criteria of the Department of Health in Taiwan, we defined obesity as BMI ≥27 kg/m^2^ [21,22]. Waist circumference ≥90 cm in men or ≥80 cm in women represented central obesity. The waist–hip ratio (WHR) was calculated by dividing the waist circumference by the hip circumference, and a WHR >0.9 and > 0.85 indicated obesity for men and women, respectively, according to the WHO-specified criteria [23]. The diagnosis of obesity was based on the BMI criteria in this study. For measurement of body fat percentage, a Tanita body composition analyzer, BC-420MA (Tanita Corp., Tokyo, Japan), was used through bioelectrical impedance analysis. Subjects were diagnosed with hypertension if the BP was ≥140/90 mmHg [24].

### 2.5 Statistical analysis

All statistical analyses were conducted using SAS version 9.4 software (SAS Institute Inc., Cary, NC). Continuous variables were described as means ± standard deviation. Comparisons of continuous variables were assessed by ANOVA and identified the significant pairs using post hoc tests. Comparisons of categorical variables were undertaken using the chi-square test. To adjust for potential confounders, the associations among *ABCG2* rs2231142, SUA levels, and risk of FRS-CVD were analyzed in the logistic regression models. When interaction terms were significant, stratified analyses of the interactive effects between *ABCG2* rs2231142 variants and BMI on SUA levels were performed while controlling confounding covariates. Odds ratios (OR) and 95 % confidence intervals (95 % CI) were calculated for the risk of increased FRS-CVD (≥10 % CVD risk). A *p*-value less than 0.05 was statistically significant.

## 3 Results

### 3.1 Demographic data and CVD risks by sex, SUA level, and *ABCG2* rs2231142 genetic variants

A total of 67,994 male and 71,514 female participants was enrolled (Table 1). Although *ABCG2* rs2231142 TT genotype was associated with HUA, we found that participants with TT group exhibited lower FRS-CVD in male participants with HUA and female participants regardless of SUA levels. Moreover, participants with TT genotypes had lower SBP, lower smoking rate, and higher HDL-C levels than their counterparts. In addition, *ABCG2* rs2231142 TT carriers had lower BMI, body fat composition, waist circumference, WHR, BUN and TG in both sexes with or without HUA (Table 2). Lower creatinine was observed in male TT carriers without HUA. Higher eGFR was noticed in female TT carriers without HUA, while lower UACR was found in female TT carriers with HUA. Lower fasting glucose was noted in female TT carriers with or without HUA. Surprisingly, TT genotypes was associated with favorable cardiometabolic profiles when compared with GG and GT genotypes.

**Table 1:** Demographic data and CVD risks by sex, SUA and *ABCG2* rs2231142 variants.

Variables	Male without HUA				Male with HUA			
Genotype	GG (n = 26542)	GT (n = 22188)	TT (n = 4673)	*p* value	GG (n = 5529)	GT (n = 6965)	TT (n = 2097)	*p* value
FRS-CVD (%)^a^	8.27 ± 6.80	8.11 ± 6.74	8.11 ± 6.71	0.022^†^	10.51 ± 7.93	10.27 ± 7.82	10.07 ± 7.78	0.067
Age (years)^a^	50.8 ± 11.1	50.8 ± 11.1	50.9 ± 11.2	0.775	49.7 ± 11.6	49.7 ± 11.6	49.6 ± 11.6	0.948
LDL-C (mg/dl)^a^	120.2 ± 31.0	120.1 ± 31.4	119.6 ± 31.1	0.493	126.5 ± 32.9	125.4 ± 32.4	126.3 ± 32.9	0.170
HDL-C (mg/dl)^a^	53.5 ± 13.1	53.9 ± 13.2	54.3 ± 13.2	<0.001^c^^d^	45.6 ± 10.4	45.8 ± 10.3	46.9 ± 11.4	<0.001^d^^e^
SBP (mmHg)^a^	120.3 ± 17.7	120 ± 17.6	119.6 ± 18.0	0.013	127.6 ± 17.0	127.2 ± 17.1	126.6 ± 16.8	0.059
DM (%)^b^	1657 (6.2)	1361 (6.1)	292 (6.2)	0.870	332 (6.3)	384 (5.5)	111 (5.3)	0.102
Smoking (%)^b^	9218 (34.7)	7335 (33.1)	1486 (31.8)	<0.001^c^^d^	2972 (56.5)	3844 (55.2)	1116 (53.2)	0.033^d^
	Female without HUA				Female with HUA			
Genotype	GG (n = 29521)	GT (n = 25504)	TT (n = 5557)	*p* value	GG (n = 4099)	GT (n = 5270)	TT (n = 1563)	*p* value
FRS-CVD (%)^a^	4.05 ± 3.87	3.91 ± 3.80	3.79 ± 3.68	<0.001^c^^d^	6.82 ± 5.18	6.59 ± 5.15	6.26 ± 5.06	<0.001^d^
Age (years)^a^	50.0 ± 10.8	49.6 ± 10.7	49.4 ± 10.7	<0.001^c^^d^	53.8 ± 10.4	53.7 ± 10.4	53.7 ± 10.4	0.922
LDL-C (mg/dl)^a^	118.8 ± 31.3	118.7 ± 31.3	118.8 ± 30.6	0.938	130.5 ± 33.7	130.1 ± 34.0	128.1 ± 32.2	0.054
HDL-C (mg/dl)^a^	59.1 ± 13.2	59.3 ± 13.1	59.6 ± 13.1	0.046	51.7 ± 11.5	52.4 ± 12.1	53.4 ± 12.8	<0.001^c^^d^^e^
SBP (mmHg)^a^	115 ± 17.5	114.8 ± 17.4	114.3 ± 17.4	0.009^d^	124.6 ± 19.0	123.8 ± 18.5	122.2 ± 18.3	<0.001^d^^e^
DM (%)^b^	1158 (3.9)	968 (3.8)	968 (3.8)	0.260	350 (8.5)	397 (7.5)	99 (6.3)	0.016^d^
Smoking (%)^b^	2978 (10.1)	2633 (10.3)	599 (10.8)	0.258	431 (10.5)	569 (10.8)	158 (10.1)	0.720

CVD, cardiovascular disease; SUA, serum uric acid; HUA, hyperuricemia (defined as serum uric acid level≥ 7.0 mg/dl for male subjects and ≥6.0 mg/dl for female subjects); FRS-CVD, Framingham risk score for cardiovascular disease; HDL-C, high density lipoprotein cholesterol; LDL-C, low density lipoprotein cholesterol; SBP, systolic blood pressure; DM, diabetes mellitus.

Bonferroni test was used for post-hoc analysis.

a Continuous variables were expressed as mean ± standard deviation (SD) and were analyzed using ANOVA comparison between groups.

b Categorical variables were expressed as numbers (percent) and were analyzed using the Chi-square test.

c Significance between GG and GT groups.

d Significance between GG and TT groups.

e Significance between GT and TT groups.

**Table 2:** Biochemical and anthropometric measurements by sex, SUA and *ABCG2* rs2231142 variants.

Variables	Male without HUA (N = 53403)					Male with HUA (N = 14591)			
	GG (n = 26542)	GT (n = 22188)		TT (n = 4673)	*p* value	GG (n = 5529)	GT (n = 6965)	TT (n = 2097)	*p* value
BMI (kg/m2)	24.2 ± 3.58	24.0 ± 3.51		23.8 ± 3.57	<0.001^a^^b^^c^	27.0 ± 3.69	26.5 ± 3.67	26.2 ± 3.67	<0.001^a^^b^^c^
Body fat percentage (%)	26.7 ± 7.54	26.9 ± 7.56		26.9 ± 7.55	0.023^†^	26.1 ± 6.02	25.7 ± 6.27	25.5 ± 6.59	<0.001^a^^b^^c^
Waist circumference (cm)	83.8 ± 9.81	83.2 ± 9.68		82.5 ± 9.77	<0.001^a^^b^^c^	91.6 ± 9.39	90.6 ± 9.35	89.5 ± 9.62	<0.001^a^^b^^c^
Waist-hip ratio	0.87 ± 0.07	0.87 ± 0.07		0.86 ± 0.07	<0.001^a^^b^^c^	0.92 ± 0.05	0.91 ± 0.06	0.90 ± 0.06	<0.001^a^^b^^c^
BUN (mg/dL)	13.3 ± 3.81	13.1 ± 3.71		13.0 ± 3.78	<0.001^a^^b^	14.7 ± 4.86	14.3 ± 4.45	14.1 ± 4.59	<0.001^a^^b^
Creatinine (mg/dL)	0.75 ± 0.32	0.74 ± 0.28		0.74 ± 0.36	<0.001^a^^b^	0.95 ± 0.47	0.94 ± 0.31	0.94 ± 0.29	0.143
eGFR (ml/min/1.73m2)	101.9 ± 14.1	102.1 ± 14.2		102.1 ± 14.7	0.191	92.9 ± 17.3	93.1 ± 17.0	92.9 ± 17.2	0.873
UACR (mg/g)	37.0 ± 231.2	34.5 ± 194.5		31.3 ± 120.5	0.234	59.3 ± 286.1	47.9 ± 336.5	48.5 ± 544.2	0.264
Fasting glucose (mg/dL)	96.8 ± 22.2	96.8 ± 23.0		96.3 ± 21.5	0.327	98.6 ± 17.9	98.2 ± 17.8	97.7 ± 19.3	0.124
Total cholesterol (mg/dL)	193.3 ± 35.0	193.4 ± 35.4		192.9 ± 35.2	0.693	198.7 ± 36.4	197.5 ± 36.5	198.8 ± 38.3	0.135
Triglyceride (mg/dL)	114.1 ± 97.0	111.4 ± 83.0		109.6 ± 84.2	<0.001^a^^b^	170.2 ± 138.0	165.7 ± 145.7	157.7 ± 142.7	0.003^b^
Uric acid (mg/dL)	5.18 ± 1.02	5.27 ± 1.02		5.33 ± 1.02	<0.001^a^^b^^c^	7.82 ± 0.79	7.96 ± 0.88	8.11 ± 0.99	<0.001^a^^b^^c^
Variables	Female without HUA (N = 60582)					Female with HUA (N = 10932)			
	GG (n = 29521)		GT (n = 25504)	TT (n = 5557)	*p* value	GG (n = 4099)	GT (n = 5270)	TT (n = 1563)	*p* value
BMI (kg/m2)	23.2 ± 3.49		23.1 ± 3.41	22.8 ± 3.32	<0.001^a^^b^^c^	26.5 ± 4.16	26.1 ± 4.31	25.6 ± 4.10	<0.001
Body fat percentage (%)	31.2 ± 6.12		31.1 ± 6.02	30.7 ± 5.92	<0.001^a^^b^^c^	36.8 ± 6.21	36.1 ± 6.49	35.4 ± 6.28	<0.001
Waist circumference (cm)	79.7 ± 9.20		79.3 ± 9.10	78.8 ± 8.94	<0.001^a^^b^^c^	87.6 ± 10.13	86.6 ± 10.47	85.7 ± 10.38	<0.001
Waist-hip ratio	0.84 ± 0.07		0.84 ± 0.07	0.84 ± 0.07	<0.001^a^^b^^c^	0.89 ± 0.06	0.88 ± 0.07	0.88 ± 0.07	<0.001
BUN (mg/dL)	12.4 ± 3.40		12.3 ± 3.33	12.1 ± 3.29	<0.001^a^^b^^c^	14.2 ± 4.76	13.9 ± 4.72	13.8 ± 4.58	0.005
Creatinine (mg/dL)	0.60 ± 0.14		0.60 ± 0.17	0.60 ± 0.12	0.030	0.69 ± 0.32	0.70 ± 0.38	0.69 ± 0.35	0.905
eGFR (ml/min/1.73m2)	106.5 ± 12.5		107.0 ± 12.2	107.3 ± 12.2	<0.001^a^^b^	96.9 ± 16.8	97.0 ± 16.3	97.4 ± 16.4	0.587
UACR (mg/g)	37.9 ± 233.9		36.0 ± 190.6	40.3 ± 278.2	0.420	81.4 ± 357.6	56.2 ± 209.1	53.9 ± 187.5	<0.001
Fasting glucose (mg/dL)	93.3 ± 18.3		92.9 ± 17.9	92.6 ± 17.0	0.010^b^	98.9 ± 19.9	98.2 ± 19.7	97.2 ± 19.9	0.011
Total cholesterol (mg/dL)	196.1 ± 35.5		195.9 ± 35.3	195.7 ± 34.8	0.690	207.3 ± 38.2	207.1 ± 38.3	205.6 ± 36.7	0.334
Triglyceride (mg/dL)	96.4 ± 66.5		95.0 ± 64.6	93.0 ± 71.8	<0.001^a^^b^	147.1 ± 118.0	142.9 ± 92.8	136.1 ± 86.9	0.001
Uric acid (mg/dL)	4.48 ± 0.8		4.56 ± 0.78	4.66 ± 0.77	<0.001^a^^b^^c^	6.70 ± 0.75	6.76 ± 0.77	6.81 ± 0.82	<0.001

SUA, serum uric acid; HUA, hyperuricemia (defined as serum uric acid level≥ 7.0 mg/dl for male subjects and ≥6.0 mg/dl for female subjects); BMI, body mass index; BUN, blood urea nitrogen; eGFR, estimated glomerular filtration rate; UACR, Urine albumin to creatinine ratio.

Continuous variables were expressed as mean ± standard deviation (SD) and were analyzed using ANOVA comparison between groups.

Bonferroni test was used for post-hoc analysis.

a Significance between GG and GT groups.

b Significance between GG and TT groups.

c Significance between GT and TT groups.

### 3.2 Association of CVD risks and SUA levels by *ABCG2* rs2231142 genotypes

**Fig. 1:**
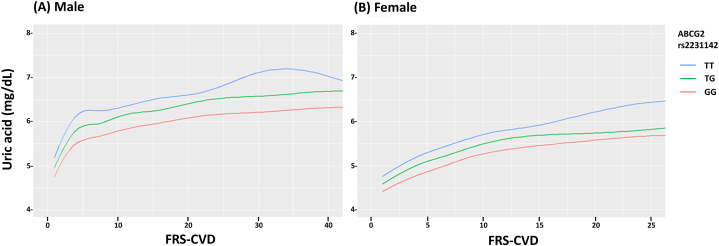
Association CVD risks and SUA levels by *ABCG2* rs2231142 genotypes in Taiwanese population. FRS-CVD, Framingham risk score for cardiovascular disease.

Fig. 1 (A) and (B) depict the association of CVD risks and SUA levels by *ABCG2* rs2231142 genotypes in the male and female populations. CVD risks were positively correlated with SUA levels. Given the same FRS-CVD risk, rs2231142 TT carriers exhibit higher SUA levels as compared with their counterparts. Interestingly, we discovered that even with the same SUA level, patients with TT genotypes had lower CVD risk than those with the GT and GG.

### 3.3 Association of CVD risks and *ABCG2* rs2231142 genotypes stratified by SUA levels, sex, and BMI

Table 3 illustrates the association between *ABCG2* rs2231142 genotypes and CVD risk, stratified by SUA levels, sex, and BMI. A significant finding was observed in females with BMI <27 and with HUA, where the TT genotype was associated with a significantly reduced risk of CVD, with an OR of 0.760 (95 % CI: 0.587–0.985; p = 0.0381). In other groups, including both males and females across various BMI categories and SUA levels, the genotypes did not show statistically significant associations with CVD risk. The analysis was adjusted for confounding factors, including age, DM, smoking status, TG, UACR, and creatinine levels. This highlights the potential protective effect of the TT genotype in a specific subgroup of females while emphasizing the complex interactions of genetic and metabolic factors influencing CVD risk in broader populations.

**Table 3:** CVD risks and *ABCG2* rs2231142 genotypes stratified by SUA levels, sex and BMI.

	Without HUA		With HUA	
Gene/SNP	OR (95 % CI)	*p* value^a^	OR (95 % CI)	*p* value^a^
Male (N = 66,723)				
BMI <27				
ABCG2 rs2231142				
GG	1		1	
GT	1.047 (0.950–1.154)	0.356	0.941 (0.796–1.111)	0.472
TT	0.940 (0.789–1.120)	0.486	0.822 (0.650–1.039)	0.101
BMI ≧ 27				
ABCG2 rs2231142				
GG	1		1	
GT	1.000 (0.841–1.188)	0.998	0.973 (0.795–1.191)	0.791
TT	1.349 (0.990–1.838)	0.058	1.211 (0.893–1.641)	0.218
Female (N = 71,514)				
BMI <27				
ABCG2 rs2231142				
GG	1		1	
GT	0.972 (0.882–1.072)	0.573	1.030 (0.867–1.223)	0.737
TT	1.043 (0.884–1.230)	0.621	0.760 (0.587–0.985)	0.038
BMI ≧ 27				
ABCG2 rs2231142				
GG	1		1	
GT	0.975 (0.810–1.173)	0.785	0.856 (0.690–1.061)	0.155
TT	0.898 (0.636–1.267)	0.540	0.961 (0.696–1.328)	0.811

SUA, serum uric acid; BMI, body mass index; HUA, hyperuricemia (defined as serum uric acid level≥ 7.0 mg/dl for male subjects and ≥6.0 mg/dl for female subjects); SNP, single nucleotide polymorphism; OR, odds ratio; 95%CI, 95 % confidence interval.

a Logistic regression adjusted by age, diabetes mellitus, smoking status, triglyceride, urinary albumin-to-creatinine ratio, creatinine.

### 3.4 Association of *ABCG2* rs2231142 variants & self-reported CAD and stroke

To further analyze the difference between ABCG2 rs2231142 variants and CVD, we examined the association of ABCG2 rs2231142 with self-reported CAD and stroke using the same database (Supplementary Table 1 and Supplementary Table 2). No significant difference was found.

## 4 Discussion

Previous genome-wide association studies (GWAS) confirmed the association of *ABCG2* rs2231142 variant with HUA [9]. This single SNP also expressed the greatest effect on gout and hyperuricemia among other SNPs on the *ABCG2* gene [15]. Hence, we hypothesized the *ABCG2* rs2231142 variant is an independent CVD risk factor in a large community-dwelling Taiwanese population. Surprisingly, the TT allele was associated independently with lower FRS-CVD in non-obese hyperuricemic female participants, which has not been reported previously. For a given SUA level, the FRS-CVD was higher in the rs2231142 GG group than in the TT group. This suggests that while TT carriers tend to have higher SUA levels, they may present a lower-risk metabolic profile compared to the GG genotype group. This difference could be explored through various factors, including drug metabolism, dietary influences, and gender differences.

ABCG2 plays a crucial role in drug metabolism [12]. Since rosuvastatin is a substrate of ABCG2, the *ABCG2* rs2231142 variant can affect rosuvastatin pharmacokinetics and its therapeutic efficacy. The mean plasma concentrations of rosuvastatin and its metabolite were higher in subjects with the *ABCG2* rs2231142 TT genotype [25]. The reduced activity of ABCG2 in rs2231142 TT allele carriers increases the absorption of rosuvastatin in the gastrointestinal tract while decreasing drug efflux in biliary ducts [26]. Rosuvastatin is known to lower LDL-C and TG while raising HDL-C levels [27]. The T allele of rs2231142 substantially increased the lipid-lowering efficiency of rosuvastatin in Asian individuals with dyslipidemia [28]. In our study, participants with the *ABCG2* rs2231142 TT genotype exhibited higher HDL-C and lower TG levels, with no difference observed in LDL-C levels. The improved lipid profile in these TT carriers may be partially due to the stronger effects of statin drugs. However, since no significant difference in LDL-C levels was observed, this suggests that other mechanisms related to this SNP may be influencing the lipid profile.

In terms of dietary impact, ABCG2 typically functions to pump various compounds, including flavonoids, out of enterocytes into the intestinal lumen, limiting their absorption and systemic availability. The *ABCG2* rs2231142 variant generally decreases the function of the ABCG2 transporter, which in turn might lead to higher blood concentrations of flavonoids [29]. Flavonoids can improve lipid profiles (by increasing HDL-C and decreasing LDL-C and TG), reduce blood pressure, and lower inflammation markers [30]. This is consistent with our findings, where participants with the ABCG2 rs2231142 TT genotype showed SBP and a better lipid profile (except for LDL-C).

Previous study linked *ABCG2* rs2231142 variant with reduced daily coffee consumption [31]. U.K. biobank data reported an U-shaped curve regarding coffee consumption and CAD risk, indicating that both very low and very high intakes may be associated with higher risks than moderate intake [32]. This may be another explanation for our result.

In terms of gender effects, estrogen exerts protective effects on the cardiovascular system through its vasodilatory and anti-inflammatory properties [33]. Additionally, estrogen has a urate-lowering effect by promoting the excretion of urate via intestinal ABCG2 [34]. There is evidence that ABCG2 is involved in lipid transport, particularly in the context of conjugated steroid hormones [35]. The rs2231142 variant may impair the transport of these hormone conjugates, potentially affecting the metabolism and clearance of estrogen in the body. This suggests that sex hormones might interact with the *ABCG2* rs2231142 variant, potentially explaining the gender-specific protective effects of this variant on CVD risk and serum uric acid (SUA) levels. Further research is necessary to confirm this hypothesis and to understand why the protective effect of the *ABCG2* rs2231142 variant on CVD risk appears to be observed primarily in females.

The rs2231142 variant may lack protective effects against CVD risk in obese individuals due to the complex interplay of metabolic disturbances, chronic inflammation, dysfunctional lipid metabolism, and elevated uric acid levels commonly associated with obesity [36,37]. The overall metabolic disruptions present in obesity could potentially negate the benefits of the rs2231142 variant. In obese individuals, the combination of genetic predisposition and environmental factors, such as diet and physical inactivity, could further diminish the variant's protective effects. Additionally, obese individuals are often on medications for conditions like hypertension, DM, and hyperlipidemia. The interactions between these medications and the ABCG2 transporter could alter the impact of the rs2231142 variant on CVD risk.

Although some studies suggest that the *ABCG2* rs2231142 variant is associated with a poor metabolic profile, our findings do not align with these results. A previous small cohort study (n = 203) reported significantly higher blood glucose and HbA1c levels in diabetic patients carrying the *ABCG2* rs2231142 variant [38]. However, our study found no significant differences in fasting glucose levels or DM prevalence between individuals with the ABCG2 rs2231142 variant and controls. Interestingly, there was a significantly lower DM prevalence among TT carriers in the hyperuricemic female group. A recent systemic review and meta-analysis concluded that rs2231142 was linked to lower levels of HDL-C, and higher levels of LDL-C and TC [28], which contradicts our findings. This discrepancy may be due to the higher heterogeneity of the meta-analysis (fifteen studies involving 34,150 individuals) compared to our study. Understanding how this variant interacts with lipid profiles is crucial to determining its impact on CVD risk.

The main finding of this study is that a novel *ABCG2* rs2231142 T allele, which is linked to HUA, is surprisingly a protective factor for CVD risk, particularly in the non-obese female population with HUA. The study has advantages of large sample size and are adjusted for traditional CAD risk factors. Our results provide a gender-specific reference nomogram for Framingham risk score prediction by uric acid level and *ABCG2* genotype in the Taiwanese population.

However, there are some limitations of our study. First, the inability to ascertain a causal relationship between *ABCG2* rs2231142 polymorphism and CVD due to the cross-sectional design of this study needs to be considered. A prospective study is needed to confirm our results. Second, the FRS is a surrogate CV outcome prediction model, which will be biased by its components. Nevertheless, after adjusting for potential confounding factors, the rs2231142 GG genotype is still independently associated with CVD risks. Third, previous studies have demonstrated that the *ABCG2* rs2231142 minor allele frequency (MAF) varies highly across different ethnic groups, with <1 % MAF in Africans, and up to 34 % in Chinese population [39]. A threefold higher prevalence of rs2231142 TT genotype was observed in East Asian populations [40] compared to European populations [9]. Thus, our result may not be extrapolated to ethnic groups other than Han Chinese. Lastly, we did not include all CVD risk factors. Alcohol consumption and physical activity were not listed in the analysis. However, previous study showed that the aforementioned factors, compared with traditional risk factors included in FRS, did not improve outcome [41]. Moreover, it would not be possible to eliminate the potential bias from concomitant medication because the information was lacking in the TWB database.

## 5 Conclusion

Our results showed that *ABCG2* rs2231142 T allele carriers were associated with lower FRS-CVD in the non-obese, hyperuricemic female population. Future study with a validated cardiovascular outcome is warranted to elucidate the relationship between *ABCG2* genetic variants and cardiovascular events.

## Ethical approval and consent to participate

This study was conducted in accordance with the Declaration of Helsinki and was approved (IRB no. CE16270B-2) by the Institutional Review Board of Taichung Veterans General Hospital. The Institutional Review Board of Taichung Veterans General Hospital waived the requirement for informed consent because the participants’ data were anonymized prior to analysis.

## Consent for publication

Not applicable.

## Availability of data and materials

The genetic data utilized in our study were obtained from the Taiwan Biobank (TWB) database, which is a government-supported, prospective cohort study encompassing a comprehensive range of phenotypic measurements and genomic data collected on the Taiwanese population [https://www.twbiobank.org.tw/new_web/index.php]. However, due to the Personal Information Protection Act implemented by the Taiwanese government in 2012, the data used in our study cannot be made publicly available in the manuscript, supplemental files, or a public repository. The TWB imposes access restrictions on the underlying data for approved reasons.

For researchers interested in accessing the data, we recommend submitting a formal proposal to obtain approval from the ethics review committee of the appropriate governmental department in Taiwan. They will provide further guidance on the data access procedure based on the specific requirements.

## Funding

This study was funded by 10.13039/100020595National Science and Technology Council, Taiwan [NTSC 111-2314-B-005-007 -MY3], and 10.13039/100010792Taichung Veterans General Hospital, Taiwan [TCVGH-1127301C, TCVGH-1127302D, TCVGH-YM1120110, TCVGH-1137310C, TCVGH-1137319C, and TCVGH-1137302D] to YMC.

## Role of the funding source

The funding sources were not involved in study design, data collection, analysis, interpretation, manuscript preparation and submission.

## Data availability statement

All data accessed and analyzed in this study are available in the article and its supplementary materials.

## CRediT authorship contribution statement

**Chun-Kang Lee:** Writing – original draft, Methodology, Conceptualization. **I-Chieh Chen:** Writing – original draft, Investigation, Formal analysis, Data curation. **Hsueh-Ju Lin:** Investigation, Formal analysis. **Ching-Heng Lin:** Supervision, Methodology, Investigation. **Yi-Ming Chen:** Writing – review & editing, Project administration, Investigation, Funding acquisition.

## Declaration of competing interest

The authors declare that they have no known competing financial interests or personal relationships that could have appeared to influence the work reported in this paper.
