# Supplementary material for: Association of the ABCG2 rs2231142 variant with the Framingham Cardiovascular Disease Risk score in the Taiwanese population
Source: Heliyon. 2024 Sep 11;10(18):e37839. doi: 10.1016/j.heliyon.2024.e37839 (PMC11417327; doi:10.1016/j.heliyon.2024.e37839)
Supplement: Multimedia component 1 [file mmc1.docx]

**Supplementary files:**

**Supplementary Table 1.** Prevalence of self-reported CAD in study subjects with and without the ABCG2 rs2231142 variants.

| Variables | GG genotype | | Self-reported CAD (%) | GT genotype | | Self-reported CAD (%) | TT genotype | | Self-reported CAD (%) | *p* value^a^ |
| --- | --- | --- | --- | --- | --- | --- | --- | --- | --- | --- |
|  | With CAD (n=581) | Without CAD (n=51959) |  | With CAD (n=554) | Without CAD (n=47546) |  | With CAD (n=134) | Without CAD (n=11025) |  |  |
| Total | 581 | 51959 | 1.11 | 554 | 47546 | 1.15 | 134 | 11025 | 1.20 | 0.6219 |
| Male | 358 | 18562 | 1.89 | 364 | 16962 | 2.10 | 93 | 3946 | 2.30 | 0.1529 |
| Female | 223 | 33397 | 0.66 | 190 | 30584 | 0.62 | 41 | 7079 | 0.58 | 0.6149 |

CAD, coronary artery disease.

^a^ The prevalence comparisons between the three groups were analyzed using the Chi-square test.

| Variables | GG genotype | | Self-reported stroke (%) | GT genotype | | Self-reported stroke (%) | TT genotype | | Self-reported stroke (%) | *p* value^a^ |
| --- | --- | --- | --- | --- | --- | --- | --- | --- | --- | --- |
|  | With Stroke (n=311) | Without Stroke (n=52229) |  | With Stroke (n=288) | Without Stroke (n=47812) |  | With Stroke (n=68) | Without Stroke (n=11091) |  |  |
| Total | 311 | 52229 | 0.59 | 288 | 47812 | 0.60 | 68 | 11091 | 0.61 | 0.9735 |
| Male | 190 | 18730 | 1.00 | 168 | 17159 | 0.97 | 45 | 3994 | 1.11 | 0.7061 |
| Female | 121 | 33499 | 0.36 | 120 | 30654 | 0.39 | 23 | 7097 | 0.32 | 0.6533 |

**Supplementary Table 2.** Prevalence of self-reported stroke in study subjects with and without the ABCG2 rs2231142 variants.

^a^ The prevalence comparisons between the three groups were analyzed using the Chi-square test.
