# Supplementary material for: Association of the ABCG2 rs2231142 variant with the Framingham Cardiovascular Disease Risk score in the Taiwanese population
Source: Heliyon. 2024 Sep 11;10(18):e37839. doi: 10.1016/j.heliyon.2024.e37839 (PMC11417327; doi:10.1016/j.heliyon.2024.e37839)
Supplement: Multimedia component 2 [file mmc2.pdf]

臺灣人體生物資料庫

健康參與者

調查問卷 V1.2

個　案　編　號： ____________________

Barcode 黏貼處：

執行單位:

中央研究院生物醫學科學研究所

中華民國一百零四年七月

面 訪

面訪開始時間：西元□□□□年/□□月/□□日/□□時/□□分 (24 小時制-由**訪員**填寫)

面訪結束時間：西元□□□□年/□□月/□□日/□□時/□□分 (24 小時制-由**訪員**填寫)

資料收集情形：第一次---訪員□□□(編號) 簽名：______________________，
本人回答 □，代答者 □(其他)______________，
自第□□□節起尋求代答，原因 □(其他)__________________，
收集情形代碼 □，尚未收完原因_________________________
第二次---訪員□□□(編號) 簽名：______________________，
本人回答 □，代答者 □(其他)______________，
自第□□□節起尋求代答，原因 □(其他)__________________，
收集情形代碼 □，尚未收完原因_________________________
第三次---訪員□□□(編號) 簽名：______________________，
本人回答 □，代答者 □(其他)______________，
自第□□□節起尋求代答，原因 □(其他)__________________，
收集情形代碼 □，尚未收完原因_________________________
(請參考【**附表一**】之代碼)

稽 核

稽 核 人 員：□□□(編號) 簽名：______________________

稽 核 時 間：西元□□□□年/□□月/□□日/□□時/□□分 (24 小時制-由**督導**填寫)

資料收集情形：□1. 良好 □2. 尚需補遺

補 遺

補 遺 時 間：西元□□□□年/□□月/□□日/□□時/□□分 (24 小時制-由**訪員**填寫)

資料收集情形：第一次---訪員□□□(編號) 簽名：______________________，
回答者 □，代答者 □(其他)______________，
自第□□□節起尋求代答，原因 □(其他)__________________，
收集情形代碼 □，尚未收完原因_________________________
第二次---訪員□□□(編號) 簽名：______________________，
回答者 □，代答者 □(其他)______________，
自第□□□節起尋求代答，原因 □(其他)__________________，
收集情形代碼 □，尚未收完原因_________________________
第三次---訪員□□□(編號) 簽名：______________________，
回答者 □，代答者 □(其他)______________，
自第□□□節起尋求代答，原因 □(其他)__________________，
收集情形代碼 □，尚未收完原因_________________________
(請參考【**附表一**】之代碼)

再 審

再 審 人 員：□□□(編號) 簽名：______________________

再 審 時 間：西元□□□□年/□□月/□□日/□□時/□□分 (24 小時制-由**督導**填寫)

資料收集情形：□1. 良好 □2. 無法補齊，原因______________________

A. 基本人口學變項

年齡、性別

A1. 您身分證上登記的出生日期為□□□□□□□□（YYYY/MM/DD，以國曆記，如 19600401）

A1a. 請問您實際上是否在那一天出生的？

□1 是 ⟶【跳問 A1c_1.】　□2 否　(□88 不知道 □99 拒答)

A1b. 您實際上的出生年月日是什麼時候？

□□□□□□□□ (YYYY/MM/DD，以國曆記，如 19601201)

(□88 不知道 □99 拒答)

請訪員自行計算:

A1c_1. 個案實際上的年齡為□□ 足歲。

A1c_2. 個案是否滿 60 歲？ □1 是　□2 否

請訪員自行計算:

A2. 受訪者性別：□1 男性　□2 女性

教育程度

A3. 您的最高學歷是什麼？

□1 未受過正規教育，不識字

□2 自修，識字

□3 小學(含肄業)

□4 國(初)中(含肄業)

□5 高中(職) (含肄業)

□6 大學(專) (含肄業)

□7 研究所及以上(含肄業)

(□88 不知道 □99 拒答)

婚姻狀況

A4. 您目前的婚姻狀況是如何？

□1 未婚

□2 已婚

□3 離婚或分居

□4 配偶已去世

(□99 拒答)

A5. 您是否獨居？

□1 否

□2 是

(□99 拒答)

籍貫及居住史

A6. 您**親生**母親的籍貫是哪裡？ **可複選**

□1 臺灣閩南人 □3 大陸各省份(請寫出省籍代號)：□□

□2 臺灣客家人 □4 臺灣原住民(請寫出族別代號)：□□

□5 其他，請說明：＿＿＿＿＿＿

(□88 不知道 □99 拒答)

A7. 您**親生**父親的籍貫是哪裡？ **可複選**

□1 臺灣閩南人 □3 大陸各省份(請寫出省籍代號)：□□

□2 臺灣客家人 □4 臺灣原住民(請寫出族別代號)：□□

□5 其他，請說明：＿＿＿＿＿＿

(□88 不知道 □99 拒答)

大陸各省籍代號 **可複選，至多兩項**

01. 山西	09. 江西	17. 浙江	25. 綏遠	33. 廣東
02. 山東	10. 安徽	18. 陝西	26. 福建	34. 廣西
03. 四川	11. 西康	19. 海南	27. 新疆	35. 遼北
04. 甘肅	12. 西藏	20. 黑龍江	28. 察哈爾	36. 遼寧
05. 安東	13. 松江	21. 湖北	29. 嫩江	37. 興安
06. 合江	14. 河北	22. 湖南	30. 寧夏	
07. 吉林	15. 河南	23. 貴州	31. 蒙古	
08. 江蘇	16. 青海	24. 雲南	32. 熱河	88 不知道 99 拒答

原住民族別代號 **可複選，至多兩項**

01. 阿美族	05. 卑南族	09. 雅美族(達悟族)	13. 撒奇萊雅族
02. 泰雅族	06. 魯凱族	10. 邵族	14. 賽德克族
03. 排灣族	07. 鄒族	11. 噶瑪蘭族	15. 拉阿魯哇族
04. 布農族	08. 賽夏族	12. 太魯閣族	16. 卡那卡那富族
			88 不知道 99 拒答

A8. 您目前的居住地是＿＿＿＿＿縣（市）＿＿＿＿＿鄉(鎮、市、區) (□99 拒答)

A9. 住了多久？□□年□□月 (□88 不知道 □99 拒答)

A10. 從您出生到現在，是否曾搬遷至其他地方居住達**半年**以上(如：就學、當兵)？

□1 否，世居於此 ⟶ **【跳問 A12.】**

□2 是，但搬遷到**其他國家**

□3 是，但範圍**在臺灣地區、金、馬、澎湖、蘭嶼等離島地區內** (□88 不知道 □99 拒答)

A11. 從您出生到現在，曾經居住過時間最久的地方為？**【居住地區範圍為臺灣地區、金、馬、澎湖、蘭嶼等離島地區】**

＿＿＿＿＿縣（市）＿＿＿＿＿鄉(鎮、市、區)

A11a. 民國□□□ 年至 □□□ 年 (□88 不知道 □99 拒答)

工作狀況

A12. 您從以前到現在是否曾工作過？(無論全職或兼職工作)

□1 從來沒有 → A12a. 請問您是：□1 家庭主婦
□2 學生
□3 其他(不曾任職者)
(□99 拒答) → 【跳問B1.】

□2 是

□99 拒答 → 【跳問B1.】

A13. 您目前是否有工作？(無論全職或兼職工作)

□1 否(包含已退休) □2 是

□99 拒答

A14. 以您的主要工作來說，請問您從以前到現在都是從事同樣的工作嗎？

□1 否 → 【跳問 A15.】

(□99 拒答) → 【跳問 A15.】

□2 是 →

A14a_1. 行業 □□□ ____________ 【編號請參照**附表二**】

A14a_2. 職位 □□□ ____________ 【編號請參照**附表三**】

A14a_3. 工作內容：____________________________________ 【請敘述工作內容】

A14a_4. 平均每週工作的時數 □□□. □ 小時 (□999 拒答)

A14a_5. 從事多久的時間？ □□年□□月 (□99 拒答)

→ 【跳問 B1.】

A15. 從以前到現在，您從事最久的一份工作是？(無論全職或兼職工作)

A15a_1. 行業 □□□ ____________ 【編號請參照**附表二**】

A15a_2. 職位 □□□ ____________ 【編號請參照**附表三**】

A15a_3. 工作內容：____________________________________ 【請敘述工作內容】

A15a_4. 平均每週工作的時數 □□□. □ 小時 (□888 不知道 □999 拒答)

A15a_5. 從事多久的時間？ □□年□□月 (□99 拒答)

B.個人健康行為

喝酒

B1. 接下來的題目想請問，您目前是否有喝酒的習慣(指 150c.c/每週，持續六個月之喝酒量)？

□1 沒有或偶爾喝酒(未達 150c.c/每週，持續六個月之喝酒量) **【跳問 B2.】**

□2 已經戒酒(無論其原因，持續六個月以上沒有喝酒)

└▶B1a. 您從幾歲開始持續喝酒？ □□歲 (□88 不知道 □99 拒答)

B1b. 到戒酒為止，您持續喝酒時間有多久(包括戒斷再喝)？ □□年□□月 (□88 不知道 □99 拒答)

B1c. 您戒幾年了(指最近一次)？ □□年□□月 (□88 不知道 □99 拒答)

□3 目前仍持續喝酒

└▶B1d. 您從幾歲開始持續喝酒？ □□歲 (□88 不知道 □99 拒答)

B1e. 目前為止您總共持續喝酒幾年？ □□年□□月 (□88 不知道□99 拒答)

吸菸

B2. 您是否曾經吸過菸？ (抽過一支以上就算)

□1 否 **【跳問 B5.】**

□2 是 (□99 拒答)

B3. 您是否曾經持續吸菸六個月以上？

□1 否 **【跳問 B5.】**

□2 是 (□99 拒答)

B4. 您目前吸菸嗎？

□1 否或偶爾(但未滿六個月以上無習慣性吸菸) **【跳問 B4n.】**

□2 已戒菸(指最近一次的戒斷，且達六個月以上無習慣性吸菸)

B4a. 您第一次持續吸菸六個月以上大約是幾歲時？ □□ 歲 (□88 不知道 □99 拒答)

B4b. 您持續吸菸大約維持多久(包括戒斷後再抽)？□□年□□月 (□88 不知道 □99 拒答)

B4c. 共戒菸過幾次？ □□次 (□88 不知道 □99 拒答)

B4d. 最後一次戒菸時是幾歲？ □□ 歲 (□88 不知道 □99 拒答)

B4e. 您是因為疾病或健康的理由戒菸的嗎？ □1 否 □2 是 (□99 拒答)

請問在您戒菸前，**最常吸菸**的那段時間，

B4f. 大約持續多久？□□年□□月 (□88 不知道 □99 拒答)

B4g. 吸菸的頻率？ □1 天 □2 週 □3 月 (□88 不知道 □99 拒答)

B4h. 吸多少包？(1 包= 20 支) □□□. □包 (□888 不知道 □999 拒答)

□3 是

B4i. 您第一次持續吸菸六個月以上大約是幾歲時？ □□ 歲 (□88 不知道 □99 拒答)

B4j. 您持續吸菸大約維持多久(包括戒斷後再抽)？□□年□□月 (□88 不知道 □99 拒答)

B4k. 目前吸菸的頻率？□1 天 □2 週 □3 月 (□88 不知道 □99 拒答)

B4l. 吸多少包？(1 包= 20 支) □□□. □包 (□888 不知道 □999 拒答)

B4m. 目前吸菸量和過去相比較，您認為目前吸菸量是增加或減少？

□1 減少

□2 差不多，沒有太大改變

□3 增加 **【跳問 B5.】** (□99 拒答)

請問在過去您**最常吸菸**的那段時間，

B4n. 大約持續多久？□□年□□月 (□88 不知道 □99 拒答)

B4o. 吸菸的頻率？ □1 天 □2 週 □3 月 (□88 不知道 □99 拒答)

B4p. 吸多少包？(1 包= 20 支) □□□. □包 (□888 不知道 □999 拒答)

(□99 拒答)

B5. 您平常是否有機會處於吸到二手菸(有人在您旁邊抽菸，而您有吸入菸)**至少五分鐘以上**的環境？

□ 1 否
□ 2 是 → (□88 不知道 □99 拒答)

B5a. 您一般是在哪些場所吸到二手菸？平均每週有幾小時會吸到？ **可複選**

□1 自己家中或住處	B5a_1 □□. □小時
□2 親友家中或住處	B5a_2 □□. □小時
□3 工作場所	B5a_3 □□. □小時
□4 其他密閉公共場所(請寫出)：__________	B5a_4 □□. □小時

(□88 不知道 □99 拒答)

吃檳榔

B6. 您是否曾經吃過檳榔？

□1 從未吃過，或只吃過一兩次而已 → **【跳問 B12.】**

□2 吃過很多次 (□99 拒答)

B7. 您是幾歲開始吃檳榔？ □□ 歲 (□88 不知道 □99 拒答)

B8. 您大約吃了多久的檳榔？ □□年□□月 (□88 不知道 □99 拒答)

B9. 您目前還有在吃檳榔嗎？ (以**最近一個月**的情形來算)

□1 完全不吃了 → B9a. 戒了□□年□□月 (□88 不知道 □99 拒答)

□2 偶爾或應酬才吃 → B9b. 這個月吃 □□□顆 (□888 不知道 □999 拒答)

□3 (幾乎)每天吃 → B9c. 平均每天吃 □□□顆 → **【跳問 B11.】** (□888 不知道 □999 拒答)

B10. 您過去是否曾有一段時間較常吃檳榔？

□1 否 **【跳問 B11.】** □2 是

B10a. 您那時候平均多久吃一次？

□1 1–3 天/每月 □3 3-5 天/每週
□2 1-2 天/每週 □4 幾乎天天吃 (□88 不知道 □99 拒答)

B10b. 平均一天吃幾顆檳榔？

□1 <10 顆 □4 31-40 顆
□2 10-20 顆 □5 41 顆以上
□3 21-30 顆 (□88 不知道 □99 拒答)

B11. 您最常吃哪一種的檳榔？

□1 檳榔子、白灰、荖葉(葉仔) □4 檳榔子、無其他配料
□2 檳榔子、紅灰、荖花(青仔) □5 其他(請寫出)____________________
□3 檳榔子、荖藤 (□88 不知道 □99 拒答)

運動

【**請注意：此題調查的是指運動**(如：散步、跑步、打拳、或跳舞…等等)**行為，勞動(農忙、家務等)不算在內。**】

B12. 您平時有沒有規律運動的習慣(指每週至少運動三次、每次三十分鐘以上)？

□1 沒有

□2 有 (□99 拒答)

B13. 過去三個月，您有沒有做過任何運動呢？

□1 沒有 **【跳問 B14.】**

□2 有 (□99 拒答)

B13a.<br>您最常做的是哪三種運動？		B13b.<br>平均每月做幾次這項運動？<br>(□88 不知道 □99 拒答)	B13c.<br>平均每次會花多少時間做這項運動？<br>(□88 不知道 □99 拒答)
1	□□□ (其他請說明)______________	□□ 次/月	□□ 時□□ 分
2	□□□ (其他請說明)______________	□□ 次/月	□□ 時□□ 分
3	□□□ (其他請說明)______________	□□次/月	□□ 時□□ 分

運動名稱及代號：

010 散步	071 外丹功	081 羽毛球	100 有氧舞蹈、跳舞機
020 慢跑	072 內丹功	082 桌球	101 土風舞、國際標準舞
030 健走	073 法輪功	083 足球	110 騎單車(或腳踏車機)
040 跳繩	074 元極舞	084 高爾夫球	120 爬山
050 游泳	075 太極拳	085 槌球	130 重量訓練(如舉重)
061 體操	076 香功	086 網球	140 爬樓梯
062 甩手運動	077 其他氣功【請寫出】	087 籃球	150 搖呼拉圈
063 瑜珈		088 其他球類【請寫出】	160 其他【請寫出】

(□999 拒答)

體重控制

B14. 您滿 18 歲以後體重最輕的時候是多少公斤？ □□□. □公斤 （□888 不知道 □999 拒答）

B15. 您滿 18 歲以後體重最重的時候是多少公斤？【懷孕時的體重不算，自產後半年開始算】

□□□. □公斤 （□888 不知道 □999 拒答）

B16. 您體重最重的時期大約是幾歲的時候？
【若最重的時期不只一個，**可複選，但 1~4 與 5 互斥**】

□1 20 歲以前
□2 20 到 30 歲
□3 31 到 50 歲
□4 超過 50 歲
□5 一直維持不變

（□88 不知道 □99 拒答）

B17. 您滿 18 歲以後曾經有過一個月內體重減少或增加超過四公斤（不含四公斤）的情形嗎？
【懷孕和生產所造成的體重變化不算】 **可複選**

□1 沒有 **【跳問 B18.】**
□2 有減少超過四公斤
□3 有增加超過四公斤

（□88 不知道 □99 拒答）

(選 2 或 3 者續答：)

B17ac. 那時您體重最重的時候是多少公斤？
□□□. □ 公斤 （□888 不知道 □999 拒答）

B17bd. 那時您體重最輕的時候是多少公斤？
□□□. □ 公斤 （□888 不知道 □999 拒答）

B18. 您現在是否有在控制體重？【包括減輕體重、增加體重、維持現狀等】

□1 減輕體重
□2 控制維持現狀
□3 增加體重
□4 沒有做任何控制

(選 3 或 4 者：) **【跳問 B19.】**

（□99 拒答）

(選 1 或 2 者續答：)

B18a. 您現在是採取哪一種方式控制體重？ **可複選**

□1 參加體重控制班
□2 多運動
□3 減少熱量攝取
□4 跳過幾餐不吃
□5 減少脂肪攝取
□6 減少或不吃肉類製品
□7 針灸
□8 使用市售減肥代餐包或減肥茶
□9 服用減肥藥
□10 服用瀉藥
□11 催吐
□12 禁食 24 小時或更久
□13 其他(請寫出)：______________

（□99 拒答）

依賴性物質使用習慣

B19. 您目前有沒有服用咳嗽糖漿、鎮靜安眠藥或是止痛藥的習慣（指每週至少一次，持續六個月以上）？

☐1 沒有 **【跳問 B21.】**

☐2 有

（☐99 拒答）

用藥種類、頻率及數量：

藥物種類		服用頻率		服用量	
B20. 您最常服用的依賴性物質種類（☐88 不知道 ☐99 拒答）	B20_.您最常服用的藥物名稱（☐88 不知道 ☐99 拒答）	B20a 頻率單位（☐99 拒答）	B20b. 次數（☐999 拒答）	B20c. 數量（☐99 拒答）	B20d 單位（☐99 拒答）
☐☐		☐1 天 ☐2 週	☐☐☐次	☐☐.☐/次	☐☐
☐☐		☐1 天 ☐2 週	☐☐☐次	☐☐.☐/次	☐☐
☐☐		☐1 天 ☐2 週	☐☐☐次	☐☐.☐/次	☐☐

◆ 依賴性物質種類

01 咳嗽糖漿　02 鎮靜安眠藥　03 止痛藥

◆ 藥劑單位代號

01 粒　02 包　03 瓶　04 C.C.　05 滴　06 克　07 其他(請寫出):________

就醫行為

B21. 當您覺得身體不舒服時（如頭痛、腹痛、腹瀉或輕微感冒），您**最常**是如何處理？

☐1 看西醫

☐2 看中醫

☐3 去藥房買藥吃

☐4 使用民俗療法或宗教治療（如刮痧、腳底按摩、收驚、神符等）

☐5 不理他，未做處理

☐6 其他【請寫出】：____________

C.生活環境

油煙

C1. 您曾有自己持續煮食超過六個月的經驗嗎？

□1 沒有 ⟶ 【跳問 C5.】

□2 有

C2. 請問您目前是否還有自己持續煮食的情形？

□1 否，已超過 6 個月以上沒有持續煮食

□2 最近 6 個月中未持續煮食

□3 是，目前仍持續煮食

C3. 從您開始煮食後，是否曾有中斷達六個月以上後再持續煮食的經驗？

□1 否

□2 是

請問您從開始持續煮食到現在，持續煮食超過六個月的經驗中，時間最長的一段情形：

	a. 煮食年齡	b. 煮食頻率	c. 以煎、炒、炸的方式烹調	d. 最常用哪種方式	e. 最常使用哪種食用油	f. 是否使用排油煙機
**C4**	1. □□ 歲至<br>2. □□ 歲<br>(□88不知道 □99 拒答)	□□1 次/天<br>□□2 次/週<br>□□3 次/月<br>(□88 不知道 □99 拒答)	□1 總是如此<br>□2 多數如此<br>□3 一半一半<br>□4 少數如此<br>□5 從未如此	□1 瓦斯(液化石油、天然氣)<br>□2 生煤、煤球或煤油<br>□3 木柴或木炭<br>□4 電鍋、電爐<br>□5 微波爐<br>□7 電磁爐<br>□6 其他：__________<br>(□88不知道 □99拒答)	□1 煮食時沒有用油<br>□2 植物油(大豆油、花生油、葵花油、橄欖油等)<br>□3 動物油(豬油、清香油)<br>□4 其他：__________<br>(□88不知道 □99拒答)	□1 否<br>□2 是，<br>↓<br>用了1.□□.□年<br>(□99 拒答)

煙、香接觸

C5. 您目前是否有機會處於接觸燒香(拜香、檀香粉、香環等)、蚊香（傳統蚊香、電蚊香、液體電蚊香）或香精（精油、薰香、芳香劑、噴霧、精油蠟燭）**至少五分鐘以上**的環境(包含家中及其他場合)？

□1 否 ⟶ 【跳問 C6.】
□99 拒答

□2 是 ↓

C5a. 您會接觸到那種煙、香？平均每週有幾小時會吸到？ **可複選**

□1 燒香（拜香、檀香粉、香環等）	C5a_1 □□□. □小時
□2 蚊香（傳統蚊香、電蚊香、液體電蚊香）	C5a_2 □□□. □小時
□3 香精（精油、薰香、芳香劑、噴霧、精油蠟燭）	C5a_3 □□□. □小時

(□88 不知道 □99 拒答)

水供應

C6. 您平日主要的飲水來源是在什麼地方？

□1 住家
□2 學校
□3 公司
□4 其他(請寫出)：____________

(□88 不知道 □99 拒答)

C7. 您目前的主要飲水種類是什麼？飲用了多少年？**【以飲水為主，不包括煮食所加水用量。】**

□1 自來水，	a. □□. □年	(□88 不知道 □99 拒答)
□2 淺井水（僅 3~5 公尺，可以直接用桶子取水），	a. □□. □年	(□88 不知道 □99 拒答)
□3 深井水（深約 30~100 公尺，與地下水層接觸），	a. □□. □年	(□88 不知道 □99 拒答)
□4 礦泉水，	a. □□. □年	(□88 不知道 □99 拒答)
□5 過濾水（包含 RO 逆滲透水、純水等），	a. □□. □年	(□88 不知道 □99 拒答)
□6 其他(山泉水等）(請說明)，____________	a. □□. □年	(□88 不知道 □99 拒答)

(□88 不知道 □99 拒答)

C7b. 承上，您平均每天會喝到多少量？ □□□□c.c. (□8888 不知道 □9999 拒答)

◆**註**： 飲水量之換算： 汽水罐：350 c.c.　一般喝水杯：200 c.c.
一般寶特瓶：600 c.c.　大容量寶特瓶：2000 c.c.

【C7. 回答 1 或 2 或 3 或 6 者回答】

→ C7c.當您飲用此主要飲水時，是喝未經煮沸過的水嗎？

□1 從未如此
□2 少數如此
□3 一半一半
□4 多數如此
□5 總是如此

(□88 不知道 □99 拒答)

D.飲食狀況

飲食特性【請根據**最近一個月內**的飲食狀況回答下列問題】

食物種類/頻率	總是如此	多數如此	一半一半	少數如此	從未如此	不吃此類食物	不知道	拒答
1. 當您吃肉類時(如豬、牛、羊、雞、鴨鵝等)時，是否連肥肉、肥油或皮一起吃？	1	2	3	4	5	6	88	99
2. 當您吃肉類或魚時，是否選擇用油烹調的方式(包括煎過再紅燒、蒸過的魚淋上油等)？	1	2	3	4	5	6	88	99
3. 當您吃蔬菜時，是否選擇用炒的方式？	1	2	3	4	5	6	88	99
4. 當您吃飯或麵時，是否會用滷汁或豬油或菜餚的湯汁拌飯、麵？	1	2	3	4	5	6	88	99
5. 當您食用豆製品時，是否選擇用炸的方式(如油豆腐、臭豆腐、炸豆皮)？	1	2	3	4	5	6	88	99
6. 當您吃麵包時，是否塗抹奶油、植物性奶油(瑪琪琳)或美乃滋？	1	2	3	4	5	6	88	99
7. 當您進餐時會另外加鹽、或沾醬油、醬油膏、椒鹽、辣椒醬等調味料嗎？	1	2	3	4	5	6	88	99
8. 您進餐時會以醬菜、豆腐乳、豆豉等佐菜配飯？	1	2	3	4	5	6	88	99
9. 當您吃點心零食時，會選擇以吃蔬菜或水果類等食物來代替高脂類點心零食(如：洋芋片、糕餅、甜甜圈)？	1	2	3	4	5	6	88	99
10. 當您吃肉類時，您會選擇吃其烹調方式是滷或烤的肉類(如：滷或烤雞腿)來代替油炸方式的肉類(如：炸雞排)？	1	2	3	4	5	6	88	99
11. 若某食品有推出低脂的產品選擇(如：低脂冰淇淋、低脂牛奶或脫脂牛奶、低脂沙拉醬)時，您會選用它來代替一般型的產品？	1	2	3	4	5	6	88	99
12. 您吃的食物會使用低鈉鹽、美味鹽、或薄鹽醬油等低鈉產品？	1	2	3	4	5	6	88	99
13. 您會盡量以吃魚或雞肉等較低油脂肉類來代替豬或牛肉等較高油脂肉類的攝取？	1	2	3	4	5	6	88	99

食物種類/頻率	總是如此	多數如此	一半一半	少數如此	從未如此	不吃此類食物	不知道	拒答
14.您會選擇吃瘦肉來代替五花肉或蹄膀嗎？	1	2	3	4	5	6	88	99
15.您會在某幾餐以吃清淡素食為主，來減少吃到如肉類等較高油脂的食物?	1	2	3	4	5	6	88	99
16.請問您一天中會至少吃兩種類蔬菜嗎？	1	2	3	4	5	6	88	99
17.當您吃家禽、畜肉類時，您會盡量少吃幾口嗎？	1	2	3	4	5	6	88	99

D18. 您平時是否有喝茶（指含茶葉成分的茶，不含花茶）的習慣（指每天至少一次以上）？

□1 否 □2 是

□99 拒答

D19. 您平時是否有喝咖啡的習慣（指每週至少三次以上）？

□1 否 □2 是

□99 拒答

素食習慣

D20. 您是否吃素或曾經吃素(全日素食，達半年以上)？

□1 否 → **【跳問 D21.】** □2 是，目前是 □3 以前曾是

□99 拒答

D20a. 您吃哪一種素食？

□1 純素(完全不食用動物性食品，如 ：蛋、奶、肉、魚)

□2 奶素(不吃動物性食品 ，但吃奶類食品)

□3 蛋素(不吃動物性食品 ，但吃蛋)

□4 奶蛋素(不吃動物性食品 ，但吃奶類和蛋)

D20b. 您吃素的習慣維持多久？ □□. □年 (□88 不知道 □99 拒答)

吃宵夜習慣

D21. 您通常一天吃幾餐正餐？ □□餐 (□99 拒答)

D21a. 在晚餐(正餐)之後、睡前一小時內，您是否有額外吃宵夜的習慣？

□1 否
□99 拒答
→【跳問 D22.】

□2 是

D21b. 您吃宵夜的頻率？

□1 一個月吃不到1次
□2 每個月吃1-3次
□3 每週吃1~3次
□4 每週吃4~6次
□5 幾乎每天都吃

(□99 拒答)

外食習慣【請根據**最近一個月內**的飲食狀況回答下列問題】

D22. 您**最近一個月內**是否有出外用餐(外食)的時候？

□1 否
□2 是，這個月1-3次
□3 是，每週吃1~3次
□4 是，每週吃4~6次
□5 是，幾乎天天外食(1餐)
□6 是，幾乎天天外食(2~3餐)

(□88 不知道 □99 拒答)

膳食補充品【請根據**最近一個月內**的飲食狀況回答下列問題】

D23. **最近一個月**來，您是否有規律地食用維生素、礦物質或其他補充劑？

□1 否
□88 不知道
□99 拒答
→【跳問E.】

□2 是，不過不規律
□3 是，相當規律

D24. 您使用的維生素、礦物質或其他補充劑為何？食用量為何？ (□88 不知道 □99 拒答)

補充劑種類名稱或品牌<br>(限填十五個中文字的長度)	食用頻率		c. 每次用量	d. ♦單位
	a. 頻率單位	b. 次數		
1.	□1 天 □2 週 □3 月	□□□	□□□. □	
2.	□1 天 □2 週 □3 月	□□□	□□□. □	
3.	□1 天 □2 週 □3 月	□□□	□□□. □	
4.	□1 天 □2 週 □3 月	□□□	□□□. □	
5.	□1 天 □2 週 □3 月	□□□	□□□. □	

◆單位:

(01)粒 (02)瓶 (03)包 (04)百 c.c. (05)茶匙 (06) c.c.
(07)滴 (08)湯匙 (09)碗 (10)克 (11)其他 (請說明：_________)

E.家族疾病史

疾病狀況

您**或**您的家人（親生父母親、有血緣關係的兄弟姊妹）是否曾被醫師診斷有下列疾病？【訪員請注意：若受訪者本身無該疾病，**仍須問**其家人之狀況】

疾病(可複選) (☐88 不知道 ☐99 拒答)		若有勾選該疾病者，請續答右邊題目	哪些家人有此疾病？(可複選) (☐88 不知道 ☐99 拒答)							
			自己		生父	生母	兄弟		姊妹	
			有	診斷日期(年/月) (☐88 不知道 ☐99 拒答)	有	有	有	兄弟患病人數	有	姊妹患病人數
過敏	☐ 1. 藥物過敏，藥名：____________________ (☐88 不知道)	→	☐	____/____	☐	☐	☐	☐☐	☐	☐☐
骨科或關節疾病	☐ 2. 骨質疏鬆症	→	☐	____/____	☐	☐	☐	☐☐	☐	☐☐
	☐ 3. 關節炎，哪一種：____________________ (如類風濕性、退化性…)	→	☐	____/____	☐	☐	☐	☐☐	☐	☐☐
	☐ 4. 痛風	→	☐	____/____	☐	☐	☐	☐☐	☐	☐☐
肺部及呼吸道疾病	☐ 5. 氣喘	→	☐	____/____	☐	☐	☐	☐☐	☐	☐☐
	☐ 6. 肺氣腫或慢性支氣管炎	→	☐	____/____	☐	☐	☐	☐☐	☐	☐☐

疾病(可複選)<br>(□88 不知道 □99 拒答)		若有勾選該疾病者，請續答右邊題目	哪些家人有此疾病？(可複選)<br>(□88 不知道 □99 拒答)							
			自己		生父	生母	兄弟		姊妹	
			有	診斷日期(年/月)<br>(□88 不知道 □99 拒答)	有	有	有	兄弟患病人數	有	姊妹患病人數
心血管疾病	□ 7. 瓣膜性心臟病	→	□	____/____	□	□	□	□□	□	□□
	□ 8. 冠心症	→	□	____/____	□	□	□	□□	□	□□
	□ 9. 心律不整	→	□	____/____	□	□	□	□□	□	□□
	□ 10. 心肌症(肥厚性或擴張性)	→	□	____/____	□	□	□	□□	□	□□
	□ 11. 先天性心臟病	→	□	____/____	□	□	□	□□	□	□□
	□ 12. 其他心臟病：________________	→	□	____/____	□	□	□	□□	□	□□
	□ 13. 高血脂症	→	□	____/____	□	□	□	□□	□	□□
	□ 14. 高血壓	→	□	____/____	□	□	□	□□	□	□□
	□ 15. 中風	→	□	____/____	□	□	□	□□	□	□□
糖尿病	□ 16. 糖尿病，哪一種：________________ (第一型、第二型)	→	□	____/____	□	□	□	□□	□	□□
消化道疾病	□ 17. 消化性潰瘍	→	□	____/____	□	□	□	□□	□	□□
	□ 18. 胃食道逆流	→	□	____/____	□	□	□	□□	□	□□
	□ 19. 大腸急躁症	→	□	____/____	□	□	□	□□	□	□□

疾病(可複選) (□88 不知道 □99 拒答)		若有勾選該疾病者，請續答右邊題目	哪些家人有此疾病？(可複選) (□88 不知道 □99 拒答)							
			自己		生父	生母	兄弟		姊妹	
			有	診斷日期(年/月) (□88 不知道 □99 拒答)	有	有	有	兄弟患病人數	有	姊妹患病人數
心理、情緒疾病	□ 20. 憂鬱症	→	□	____/____	□	□	□	□□	□	□□
	□ 21. 躁鬱症	→	□	____/____	□	□	□	□□	□	□□
	□ 22. 產後憂鬱症【男性不問】	→	□	____/____		□			□	□□
	□ 23. 強迫症	→	□	____/____	□	□	□	□□	□	□□
	□ 24. 酒癮或藥物濫用	→	□	____/____	□	□	□	□□	□	□□
	□ 25. 精神分裂症	→	□	____/____	□	□	□	□□	□	□□
神經系統疾病	□ 26. 癲癇	→	□	____/____	□	□	□	□□	□	□□
	□ 27. 偏頭痛	→	□	____/____	□	□	□	□□	□	□□
	□ 28. 多發性硬化症	→	□	____/____	□	□	□	□□	□	□□
	□ 29. 巴金森氏症	→	□	____/____	□	□	□	□□	□	□□
	□ 30. 失智症	→	□	____/____	□	□	□	□□	□	□□

<table>
<tr><th colspan="2" rowspan="3">疾病(可複選)
(□88 不知道 □99 拒答)</th><th rowspan="3">若有勾選該疾病者，請續答右邊題目</th><th colspan="9">哪些家人有此疾病？(可複選)
(□88 不知道 □99 拒答)</th></tr>
<tr><th colspan="2">自己</th><th>生父</th><th>生母</th><th colspan="2">兄弟</th><th colspan="2">姊妹</th></tr>
<tr><th>有</th><th>診斷日期(年/月)
(□88 不知道 □99 拒答)</th><th>有</th><th>有</th><th>有</th><th>兄弟患病人數</th><th>有</th><th>姊妹患病人數</th></tr>
<tr><td rowspan="4">其他疾病</td><td>□ 31. 肝膽結石</td><td>→</td><td>□</td><td>____/____</td><td>□</td><td>□</td><td>□</td><td>□□</td><td>□</td><td>□□</td></tr>
<tr><td>□ 32. 腎結石</td><td>→</td><td>□</td><td>____/____</td><td>□</td><td>□</td><td>□</td><td>□□</td><td>□</td><td>□□</td></tr>
<tr><td>□ 33. 腎衰竭(曾有洗腎者)</td><td>→</td><td>□</td><td>____/____</td><td>□</td><td>□</td><td>□</td><td>□□</td><td>□</td><td>□□</td></tr>
<tr><td>□ 34. 眩暈</td><td>→</td><td>□</td><td>____/____</td><td>□</td><td>□</td><td>□</td><td>□□</td><td>□</td><td>□□</td></tr>
</table>

您的家人（親生父母親、有血緣關係的兄弟姊妹）是否曾被醫師診斷有任何癌症？

<table>
<tr><th colspan="2" rowspan="3">疾病
(□88 不知道 □99 拒答)</th><th rowspan="3">若有勾選該疾病者，請續答右邊題目</th><th colspan="6">哪些家人有此疾病？(可複選)
(□88 不知道 □99 拒答)</th></tr>
<tr><th>生父</th><th>生母</th><th colspan="2">兄弟</th><th colspan="2">姊妹</th></tr>
<tr><th>有</th><th>有</th><th>有</th><th>兄弟患病人數</th><th>有</th><th>姊妹患病人數</th></tr>
<tr><td rowspan="8">原位癌症</td><td>□ 35. 肝癌</td><td>→</td><td>□</td><td>□</td><td>□</td><td>□□</td><td>□</td><td>□□</td></tr>
<tr><td>□ 36. 肺癌</td><td>→</td><td>□</td><td>□</td><td>□</td><td>□□</td><td>□</td><td>□□</td></tr>
<tr><td>□ 37. 乳癌</td><td>→</td><td>□</td><td>□</td><td>□</td><td>□□</td><td>□</td><td>□□</td></tr>
<tr><td>□ 38. 胃癌</td><td>→</td><td>□</td><td>□</td><td>□</td><td>□□</td><td>□</td><td>□□</td></tr>
<tr><td>□ 39. 大腸直腸癌</td><td>→</td><td>□</td><td>□</td><td>□</td><td>□□</td><td>□</td><td>□□</td></tr>
<tr><td>□ 40. 鼻咽癌</td><td>→</td><td>□</td><td>□</td><td>□</td><td>□□</td><td>□</td><td>□□</td></tr>
<tr><td>□ 41. 前列腺癌【女性不問】</td><td>→</td><td>□</td><td></td><td>□</td><td>□□</td><td></td><td></td></tr>
<tr><td>□ 42. 其他癌症：
________________</td><td>→</td><td>□</td><td>□</td><td>□</td><td>□□</td><td>□</td><td>□□</td></tr>
</table>

E43. 下面我會提到一些身體上的疼痛，請您告訴我**最近三個月**，您有沒有這方面的疼痛？

【若有疼痛，續問：是常常會痛或偶爾會痛呢？】

【請注意：有頭痛、偏頭痛者請加問 5a-5d，未停經之女性加問 6. 經痛】

身體上的疼痛	勾選該項目者，續答右邊題目	疼痛頻率		
		常常會痛	偶爾會痛	拒答
☐1. (全身)關節疼痛或僵硬	→	1	2	99
☐2. 頸部酸痛	→	1	2	99
☐3. 下背部疼痛、腰痛	→	1	2	99
☐4. 坐骨神經痛	→	1	2	99
☐5. 頭痛、偏頭痛【續問 5a-5d】	→	1	2	99
5_a.當您頭痛時，頭痛是否會影響到您的工作，學習或日常生活? ☐1 會 ☐2 不會<br>5_b.當您頭痛時，頭痛的程度是? ☐1 輕度 ☐2 中度 ☐3 重度<br>5_c.當您頭痛時，是否會噁心或反胃? ☐1 會 ☐2 不會<br>5_d.當您頭痛時，是否會覺得光線特別刺眼? ☐1 會 ☐2 不會				
☐6. 經痛【女性加問，已停經者不需詢問】	→	1	2	99
☐7. 其他(說明疾病或症狀)<br>其他(1)：_______________	→	1	2	99
☐其他(2)：_______________	→	1	2	99
☐其他(3)：_______________	→	1	2	99
☐其他(4)：_______________	→	1	2	99

眼部疾病

您是否曾被醫生診斷有下列的視力問題或是眼睛方面的疾病？【逐項詢問】【若有接受眼部治療，請回答接受**矯正或治療前**的情形】

視力問題	若有勾選該項目者，請續答右答右邊題目	哪隻眼睛有此病症							
		E44. 左眼				E45. 右眼			
		否	是	不知道	拒答	否	是	不知道	拒答
☐A. 白內障	→	1	2	88	99	1	2	88	99
☐B. 青光眼	→	1	2	88	99	1	2	88	99
☐C. 乾眼症	→	1	2	88	99	1	2	88	99
☐D. 視網膜剝離	→	1	2	88	99	1	2	88	99
☐E. 飛蚊症	→	1	2	88	99	1	2	88	99
☐F. 失明	→	1	2	88	99	1	2	88	99
☐G. 色盲	→	1	2	88	99	1	2	88	99
☐H. 其他(請說明是什麼視力問題或眼部疾病)<br>(1)____________<br>(2)____________	→	1	2	88	99	1	2	88	99

Patient Health Questionnaire

請您盡量回憶在**過去兩個星期內**，有多少時候您受到以下的問題所困擾?

PHQ-4	完全不會	幾天	一半以上的天數	幾乎每天	不知道	拒答
1. 感到緊張，不安或煩躁	0	1	2	3	88	99
2. 無法停止或控制憂鬱	0	1	2	3	88	99
3. 做事時提不起勁或沒有樂趣	0	1	2	3	88	99
4. 感到心情低落、沮喪或絕望	0	1	2	3	88	99

F.女性相關問題

接下來的問題，主要是想收集台灣地區女性生理、生育等相關資料，作為女性相關疾病的研究參考。我們保證維護您的隱私，且保證您所回答的內容絕對保密，請您能配合並詳細地根據您真實的情況做答。謝謝您！

F1. □1 男性不回答 ⟶ 【F 大項完成】

F2. 您是幾歲開始來月經的？ □□歲 (□88 不知道 □99 拒答)

F3. 您的月經週期一般來說規律嗎？

□1 規律 ⟶ F3a. 您的月經間隔多久來一次？ □□□天

(指前次月經開始的第一天距這次開始的第一天所經過的天數)

(□888 不知道 □999 拒答)

□2 不規律

(□99 拒答)

F4. 您目前是否仍有月經？

□1 否 ⟶ F4a. 您是幾歲停經的？ □□歲

(指最後一次來月經後，間隔12個月以上未再有月經)

(□88 不知道 □99 拒答)

F4b. 請問您停經的原因是？

□1. 自然停經

□2. 手術後停經

□3. 其他(請寫出)：______________

□ 2 是

(□88 不知道 □99 拒答)

(□99 拒答)

生育的狀況

F5. 您是否曾經懷孕過？

□1 否
□88 不知道
□99 拒答
→【跳問F9.】

□2 是 → F5a. 一共懷孕過幾次？ □□次 (□88 不知道 □99 拒答)

F5b. 第一次懷孕時幾歲？ □□歲 (□88 不知道 □99 拒答)

F5c. 最後一次懷孕時幾歲？ □□歲 (□88 不知道 □99 拒答)

F6. 您是否曾經生產過？(包括死產，不包括流產)

□1 否

□2 是 → F6a. 曾經生產過幾次？ □□次 (□88 不知道 □99 拒答)

F6b. 生第一胎時幾歲？ □□歲 (□88 不知道 □99 拒答)

F6c. 生最後一胎時幾歲？ □□歲 (□88 不知道 □99 拒答)

(□99 拒答)

F7. 您是否曾經流產過(包括自然流產及人工流產)？

□1 否

□2 是 → F7a. 曾經流產過幾次？ □□次 (□88 不知道 □99 拒答)

F7b. 請問您自然流產過幾次？ □□次 (□88 不知道 □99 拒答)

F7c. 請問您人工流產過幾次？ □□次 (□88 不知道 □99 拒答)

(□88 不知道 □99 拒答)

F8. 您曾經餵過母奶嗎？

□1 否

□2 是 → F8a. 曾經餵過幾胎母奶？ 共□□胎 (□88 不知道 □99 拒答)

(□99 拒答)

F8a._1. 生育胎數	餵到小孩幾個月大？(□88 不知道 □99 拒答)
a. 第一胎	□□. □
b. 第二胎	□□. □
c. 第三胎	□□. □
d. 第四胎	□□. □
e. 第五胎	□□. □
f. 第六胎	□□. □
g. 第七胎	□□. □
h. 第八胎	□□. □

服藥、補充劑的狀況

F9. 您是否曾因為避孕、更年期症候群、疾病治療(如:子宮內膜異位、不孕症)等因素，規律地使用荷爾蒙類西藥達半年以上？

□1 否 → 【跳問F11.】
□88 不知道 → 【跳問F11.】
□99 拒答 → 【跳問F11.】
□2 是

F10. 您曾規律地使用荷爾蒙類西藥的原因是？ **可複選**

□1 避孕，	a. 共使用 □□年□□月	(□88 不知道 □99 拒答)
□2 更年期症候群，	b. 共使用 □□年□□月	(□88 不知道 □99 拒答)
□3 調經，	c. 共使用 □□年□□月	(□88 不知道 □99 拒答)
□4 安胎，	d. 共使用 □□年□□月	(□88 不知道 □99 拒答)
□6 疾病治療，	e. 共使用 □□年□□月	(□88 不知道 □99 拒答)
□5 其他(請說明)：______________，	f. 共使用 □□年□□月	(□88 不知道 □99 拒答)

(□88 不知道 □99 拒答)

F11. 您是否曾因為安胎、調養、調經、更年期等因素，規律地使用女性調理類中藥達三個月以上？

□1 否 → 【跳問F13.】
□88 不知道 → 【跳問F13.】
□99 拒答 → 【跳問F13.】
□2 是

F12. 您曾規律地使用女性調理類中藥的原因是？ **可複選**

□1 安胎(例如：安胎十三味)，	a. 共使用□□年□□月	(□88 不知道 □99 拒答)
□2 調養(例如：八珍湯、十全大補湯)，	b. 共使用□□年□□月	(□88 不知道 □99 拒答)
□3 調經(例如：四物湯、中將湯)，	c. 共使用□□年□□月	(□88 不知道 □99 拒答)
□4 更年期症候群，	d. 共使用□□年□□月	(□88 不知道 □99 拒答)
□5 其他(請說明)：_______________，	e. 共使用□□年□□月	(□88 不知道 □99 拒答)

(□88 不知道 □99 拒答)

F13. 您是否曾規律地使用雌激素、胎盤素、月見草油等補充劑達三個月以上？**可複選，但1與2~4互斥**

□1 都沒有使用或不規律

□2 雌激素， a. 共使用□□年□□月 (□88 不知道 □99 拒答)

□3 胎盤素， b. 共使用□□年□□月 (□88 不知道 □99 拒答)

□4 月見草油， c. 共使用□□年□□月 (□88 不知道 □99 拒答)

F14. 您或您的家人（親生母親、有血緣關係的姐妹）是否曾被醫師診斷有下列女性疾病？**可複選，但 1~8 與 9 互斥**

疾病(可複選) (□88 不知道 □99 拒答)	若有勾選該疾病者，請續答右邊題目	哪些家人有此疾病？(可複選) (□88 不知道 □99 拒答)				
		**自己**		**生母**	**姊妹**	
		有	診斷日期(年/月) (□88 不知道 □99 拒答)	有	有	請填寫**姊妹中**患病人數
□ 1.嚴重經痛	→	□	____/____	□	□	□□
□ 2.子宮肌瘤	→	□	____/____	□	□	□□
□ 3.卵巢囊腫(卵巢水瘤)	→	□	____/____	□	□	□□
□ 4.子宮內膜異位 (巧克力囊腫/肌腺症)	→	□	____/____	□	□	□□
□ 5.子宮/子宮頸瘜肉	→	□	____/____	□	□	□□
□ 6.子宮癌	→			□	□	□□
□ 7.子宮頸癌	→			□	□	□□
□ 8.卵巢癌	→			□	□	□□
□ 9.以上皆無						

H. 經濟狀況

收入

H1. 請問過去一年您個人平均每個月收入大約多少錢(如：薪資、紅利、加班費、自營收入、退休金)？

□(1)沒有收入　□(7) 5-6(含)萬元　□(13) 11-12(含)萬元　□(19) 17-18(含)萬元
□(2) 1 萬元以下　□(8) 6-7(含)萬元　□(14) 12-13(含)萬元　□(20) 18-19(含)萬元
□(3) 1-2(含)萬元　□(9) 7-8(含)萬元　□(15) 13-14(含)萬元　□(21) 19-20(含)萬元
□(4) 2-3(含)萬元　□(10) 8-9(含)萬元　□(16) 14-15(含)萬元　□(22) 20 萬元以上
□(5) 3-4(含)萬元　□(11) 9-10(含)萬元　□(17) 15-16(含)萬元
□(6) 4-5(含)萬元　□(12) 10-11(含)萬元　□(18) 16-17(含)萬元

(□88 不知道 □99 拒答)

H2. 請問過去一年您全家包括所有與您同住者平均每個月收入大約多少錢(如：薪資、紅利、加班費、自營收入、退休金)？

□(1)沒有收入　□(7) 5-6(含)萬元　□(13) 11-12(含)萬元　□(19) 17-18(含)萬元
□(2) 1 萬元以下　□(8) 6-7(含)萬元　□(14) 12-13(含)萬元　□(20) 18-19(含)萬元
□(3) 1-2(含)萬元　□(9) 7-8(含)萬元　□(15) 13-14(含)萬元　□(21) 19-20(含)萬元
□(4) 2-3(含)萬元　□(10) 8-9(含)萬元　□(16) 14-15(含)萬元　□(22) 20 萬元以上
□(5) 3-4(含)萬元　□(11) 9-10(含)萬元　□(17) 15-16(含)萬元
□(6) 4-5(含)萬元　□(12) 10-11(含)萬元　□(18) 16-17(含)萬元

(□88 不知道 □99 拒答)

I. 中醫體質問項

接下來的問題, 主要是想收集中醫體質方面相關的問題，作為中醫體質相關的研究參考。
此部分約會佔用您 10-15 分鐘的時間，您有拒答的權利。

0. 請問您是否願意回答此部份的問題？
1. 否【**問卷結束，由訪員自填 J 部分**】　　　　2. 是

請您儘量回憶過去一個月內，身體是否有下列現象？請在下面各題☐中以（V）選擇此現象出現的強度或頻率。在本部分所指過去一個月內，係指從今天往前算三十天內。

	完全不會 0	稍微會 25	中等程度會 50	很會 75	最嚴重會 100
1. 雖然沒有感冒，我喉嚨中會有痰。--------------------------------	☐	☐	☐	☐	☐
2. 我會覺得手心、腳掌心或身體會熱熱的。----------------------	☐	☐	☐	☐	☐
3. 我會覺得自己怕冷、手腳冰冷或需穿比較多的衣服。-------	☐	☐	☐	☐	☐
4. 我會覺得頭重、頭昏不舒服。------------------------------------	☐	☐	☐	☐	☐
5. 我會覺得疲倦或無力不想動。------------------------------------	☐	☐	☐	☐	☐
6. 我會覺得口中黏黏的或口水黏稠。----------------------------	☐	☐	☐	☐	☐
7. 我會覺得身體或下半身重重的。-------------------------------	☐	☐	☐	☐	☐
8. 突然站起來時，我會覺得眼前發黑。-------------------------	☐	☐	☐	☐	☐
9. 我會覺得疲倦不想說話或沒力氣說話。----------------------	☐	☐	☐	☐	☐
10. 我的舌頭或口腔會破。---	☐	☐	☐	☐	☐
11. 我會覺得眼睛乾澀，或看東西不清楚。----------------------	☐	☐	☐	☐	☐
12. 我的胸、腹部或四肢會悶痛不舒服。----------------------------	☐	☐	☐	☐	☐
13. 我的胸、腹部或四肢會刺痛不舒服。----------------------------	☐	☐	☐	☐	☐
14. 我會覺得睡眠時間夠，但仍想睡覺或睡不飽。--------------	☐	☐	☐	☐	☐
15. 我會覺得氣不夠，需要深呼吸。-------------------------------	☐	☐	☐	☐	☐
16. 我的身體或手腳會有麻木感。----------------------------------	☐	☐	☐	☐	☐
17. 我會覺得胸口悶悶或緊緊的，好像有東西壓著。-----------	☐	☐	☐	☐	☐
18. 我會耳鳴。---	☐	☐	☐	☐	☐
19. 我的皮膚會無故出現瘀血烏青。-------------------------------	☐	☐	☐	☐	☐
20. 我的皮膚會乾燥、龜裂、變厚或變硬。----------------------	☐	☐	☐	☐	☐

	完全不會 0	稍微會 25	中等程度會 50	很會 75	最嚴重會 100
21. 我的身體或四肢會看到扭曲變形的血管(靜脈曲張)。------	☐	☐	☐	☐	☐
22. 我會感到腰部、膝蓋或足跟酸軟、疼痛、無力或發冷。--	☐	☐	☐	☐	☐
23. 沒有劇烈運動時，我會抽筋。----------------------------------	☐	☐	☐	☐	☐
24. 我會覺得呼吸深度短淺或喘。----------------------------------	☐	☐	☐	☐	☐
25. 我的身體側面或兩側上腹肋骨處（位置見圖中打×範圍）會有悶、脹或疼痛的感覺。 ----------------------------	☐	☐	☐	☐	☐

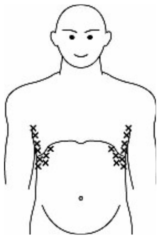

	完全不會 0	稍微會 25	中等程度會 50	很會 75	最嚴重會 100
26. 我會口渴、嘴巴乾、嘴唇乾燥龜裂，且喝水後很快又口渴。	☐	☐	☐	☐	☐
27. 我的四肢、身體、臉部或眼睛周圍會浮腫。------------------	☐	☐	☐	☐	☐
28. 平躺時我的呼吸會變喘。---	☐	☐	☐	☐	☐
29. 我的臉頰會發紅。(部位請見附圖） ------	☐	☐	☐	☐	☐

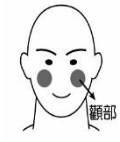

	完全不會 0	稍微會 25	中等程度會 50	很會 75	最嚴重會 100
30. 我會覺得喉嚨乾，但嘴巴或嘴唇不會乾燥。----------------	☐	☐	☐	☐	☐
31. 姿勢改變時，我會覺得天旋地轉。----------------------------	☐	☐	☐	☐	☐
32. 我覺得我的聽力減退。---	☐	☐	☐	☐	☐
33. 我吹到風會不舒服。--	☐	☐	☐	☐	☐
34. 我的舌苔會厚厚或黏黏的。-------------------------------------	☐	☐	☐	☐	☐
35. 我會覺得身體或頭面突然一陣熱熱的。-----------------------	☐	☐	☐	☐	☐
36. 我會喜歡喝溫熱的東西。--	☐	☐	☐	☐	☐
37. 沒有劇烈運動時，我的腰部會容易扭傷。--------------------	☐	☐	☐	☐	☐

	從來沒有 0	偶爾有 25	一半有一半沒有 50	常常有 75	一直都有 100
38. 除了早上第一次解尿外，我的小便顏色深黃或茶色。--------	☐	☐	☐	☐	☐
39. 我的小便量少。---	☐	☐	☐	☐	☐
40. 我的大便乾硬。---	☐	☐	☐	☐	☐
41. 我的大便不成形（腹瀉）。--	☐	☐	☐	☐	☐
42. 我會覺得嘴巴淡淡的沒有味道。--------------------------------	☐	☐	☐	☐	☐
43. 沒有喝很多水，我仍覺得小便量多。---------------------------	☐	☐	☐	☐	☐
44. 我在天亮前會因拉肚子而起床。----------------------------------	☐	☐	☐	☐	☐

＜附表二＞臺灣地區行業標準分類表

0 農、林、漁、牧、狩獵業

001 農、牧、狩獵業
002 林業及伐木業
003 漁業

1 礦業及土石採取業

011 煤礦業
012 石油、天然氣及地熱礦業
013 金屬礦業
014 鹽業
015 土礦及石礦業
016 化學及肥料礦業
017 其他礦業（如寶石礦業）
018 土石採業

2／3 製造業

020 食品製造業
021 飲料及菸草製造業
022 紡織業
023 成衣及服飾品製造業(製帽)
024 皮革、毛皮、及其他製品製造業（皮鞋製造）
025 木竹製品及非金屬家俱製造業
026 造紙、紙製品及印刷出版業
027 化學材料製造業
028 化學製品製造業
029 石油及煤製品製造業
030 橡膠製品製造業
031 塑膠製品製造業
032 非金屬礦物製品製造業（如玻璃、水泥）
033 金屬基本工業（包含廢五金、解體廢船）
034 金屬製品製造業（包含鐵窗、鋁門窗、車床製品如螺絲釘）
035 機械設備製造修配業（包含製造車床）
036 電力及電子機械器材製造修配業（包含電池製造）
037 運輸工具製造修配業
038 精密器械製造業（包含鐘錶、醫療器械）
039 雜項工業製品製造業（包含組合器具、半成品之組合如珠寶樂器等）

4 水電燃氣業

041 電力供應業
042 氣體燃料供應業
043 暖氣及熱水供應業
044 自來水供應業

5 營造業

051 土木工程業（房屋建築）
052 電路及管道工程業（包含水電行）
053 油漆、粉刷、裱蓆業
059 其他營造業

6 商業

061 批發業（如萬客隆）
062 零售業（包含電腦買賣、藥局、書局以及超商百貨）
063 國際貿易業
064 餐旅業(包含飲食業及旅館業)

7 運輸、倉儲、及通信業

071 運輸業（包括運輸服務業；如計程車、報關行及旅行社等）

072 倉儲業

073 通信業（電信業及郵政業）

8 金融、保險、不動產、及工商服務業

081 金融業 (包含證券業及典當)

082 保險業

083 經紀業
（包含不動產、市場管理）

084 法律及工商服務業
（包含顧問服務、土地代書、資訊服務維護、廣告業、及產品包裝設計、保全、影印、職業介紹）

085 機械設備租賃業

086 股票投資（包含個人投資股票）

9 公共行政、社會服務及個人服務業

090 公共行政服務業（包含看守所管理員）

091 國防事業

092 環境衛生服務業

093 教育、學術（含社會教育：如圖書館、藝術館、天文台…等）

094 大眾傳播事業

095 醫療保健服務業

096 社會福利、人民團體及其他社會服務業（包含政黨、傳教機構）

097 文化及康樂服務業（含藝術表演、文學、，如文學著作、翻譯、雕刻）

098 個人服務業、修理、洗染

099 國際機構及外國駐在機構

O 其他不能歸類之行業

100 其他不能歸類之行業
（如家庭主婦、學生、目前無工作者）

N 888 不知道

R 999 拒答

＜附表三＞臺灣地區職位分類表及說明

管理人員：監督

110 雇主與總經理（含董監事、董事長、郵電總局長、監察人、副總經理）
120 主管或經理
130 校長
140 民意代表
370 辦公室監督（如股長、科長、課長、副理、襄理）

實務工作者：學識技術層級

自願役軍人
060 將官
061 校官
062 尉官
063 士官
064 士兵

預備役軍人
065 尉官
066 士官
067 士兵

專業人員（含工程師）
201 大專教師與研究人員
202 中小學、學前特教教師

211 法學（律）專業人員類（如律師、法官）
212 語文、文物管理專業人員(如作家、記者編輯、圖書館管理)
213 藝術、娛樂(如聲樂家)
214 宗教(有神職，如神父)

221 醫師
222 藥師
223 護士、助產師、護理師

230 會計師及商業專業人員（如投資分析師、專利顧問）

240 農學生物專業人員（如農業技師）

250 工程師(含建築、資訊、測量師、技師)

助理(半)專業人員（含技術員）
301 助教
302 研究助理（不含行政總務）
303 補習班、訓練班教師（練）
311 法律、行政半專業助理（含海關、稅收檢驗員）
312 社工員、輔導員
313 半專業（如餐廳歌手、模特兒、廣告流行設計）
314 半專業（沒有神職）

321 醫療技術人員（如無照護士、檢驗師、接骨推拿、藥劑生）

322 運動半專業（如裁判、職業選手、教練）

331 會計、計算半專業助理
332 專技銷售、仲介等半專業服務（如工商業推銷、直銷員、拍賣、鑑定、採購、拉保險、勞工承辦人、經紀人、報關代理）

340 農業生物技術員或助理（含推廣人員）
350 工程技術員（含聲光、檢驗，廣電設備管制，技術獅、攝影師）
351 電子機械技術員
352 化學冶金技術員
353 採礦技術員
354 工業工程技術員
355 製圖員
359 其他技術員（含聲光、檢驗、廣電設備管制、技術師、攝影師）
360 航空､航海技術人員(如飛機駕駛）

事務性工作人員與其他類似技術層級者
410 辦公室事務性工作（如法律、行政事務性助理、打字、文書、登錄、郵運、圖書、複印、財稅事務）
420 顧客服務性事務（如櫃臺接待、其他接待、總機、掛號、旅遊事務）
431 會計（含簿記、證券）事務
432 出納事務（含售票、收費、櫃臺金融服務）
511 旅運服務生(員)(含嚮導）
512 餐飲服務生
513 廚師（含調飲料、飲食攤廚師）
514 家事管理（如管家）
515 理容整潔
516 個人照顧（如保母、陪病、按摩）
520 保安工作（如警察）
531 商店售貨（含展售）
532 固定攤販與市場售貨
610 農林木工作人員
620 漁民（含漁船駕駛）

710 營建採礦技術工（如水泥匠、板模、油漆、裝潢、水電工）
720 金屬機械技術工（如裝修機械、鐵匠焊接、板金、試車工）
790 其他技術工（如裁縫、修鞋匠、木匠、麵包師傅、手工藝、手作印刷）
810 農機械操作半技術工（如操作除草、噴藥機）
820 工業操作半技術工（如操作鑽孔機、紡織機、熔爐、發電、製藥設備）
830 組裝（配）半技工（如裝配機件、塑膠、紡織、紙、木製品）
840 車輛駕駛及移運設備操作半技術工（含船面水手）

非技術工
910 工友、小妹
920 看門（如門房、收票、帶位電梯服務員、廟公、建築物管理員、寄物管理員）
930 售貨小販（沒店面）
940 清潔工（洗車、擦鞋、洗菜、洗碗、家庭清潔工、清道、廢棄物蒐集）
950 生產體力非技術工（如挖溝體力工、手作包裝、捆紮線、封籤、簡單組裝體力工）
960 搬送非技術工（含送件、送報、搬運、球童、販賣機收款、抄表）

無正式工作者

068 學生
069 家庭主婦
070 失業
071 其他無職業者

888 不知道

999 拒答
